# A survey of foot problems in community-dwelling older Greek Australians

**DOI:** 10.1186/1757-1146-4-23

**Published:** 2011-10-20

**Authors:** Patricia Kaoulla, Nicoletta Frescos, Hylton B Menz

**Affiliations:** 1 Department of Podiatry, Faculty of Health Sciences, La Trobe University, Bundoora, Victoria, Australia; 2 Musculoskeletal Research Centre, Faculty of Health Sciences, La Trobe University, Bundoora, Victoria, Australia

## Abstract

**Background:**

Foot problems are common in older people and are associated with impaired mobility and quality of life. However, the characteristics of foot problems in older Australians for whom English is a second language have not been evaluated.

**Methods:**

One hundred and four community-dwelling people aged 64 to 90 years with disabling foot pain (according to the case definition of the Manchester Foot Pain and Disability Index, or MFPDI) were recruited from four Greek elderly citizens clubs in Melbourne, Australia. All participants completed a Greek language questionnaire consisting of general medical history, the Medical Outcomes Study Short-Form 36 (SF-36) questionnaire, the MFPDI, and specific questions relating to foot problems and podiatry service utilisation. In addition, all participants underwent a brief clinical foot assessment.

**Results:**

The MFPDI score ranged from 1 to 30 (median 14), out of a total possible score of 34. Women had significantly higher total MFPDI scores and MFPDI subscale scores. The MFPDI total score and subscale scores were significantly associated with most of the SF-36 subscale scores. The most commonly reported foot problem was difficulty finding comfortable shoes (38%), and the most commonly observed foot problem was the presence of hyperkeratotic lesions (29%). Only 13% of participants were currently receiving podiatry treatment, and 40% stated that they required more help looking after their feet. Those who reported difficulty finding comfortable shoes were more likely to be female, and those who required more help looking after their feet were more likely to be living alone and have osteoarthritis in their knees or back.

**Conclusions:**

Foot problems appear to be common in older Greek Australians, have a greater impact on women, and are associated with reduced health-related quality of life. These findings are broadly similar to previous studies in English-speaking older people in Australia. However, only a small proportion of this sample was currently receiving podiatry treatment, and a substantial number stated that they required more help looking after their feet. To address this issue, steps need to be taken to increase awareness of podiatry services among older Greek Australians.

## Background

It is now well established that foot problems are highly prevalent in older people [1-7], and have a significant detrimental impact on mobility and quality of life in this age-group [2,8-10]. Foot problems such as hallux valgus, lesser toe deformity and plantar hyperkeratotic lesions frequently result in pain, affect walking speed and balance, and increase the risk of falls [1,11-13]. Furthermore, older people with foot problems exhibit significantly lower scores on health-related quality of life questionnaires [9,14,15], indicating that the impact of foot disorders extends well beyond localised pain and discomfort.

One of the major limitations of the available literature pertaining to foot disorders in older people is that it has focused almost exclusively on English-speaking populations, primarily because survey documents have not been developed or validated in other languages. This is a particular problem in countries with large ageing migrant populations such as Australia. The 2006 Australian census [16] revealed that 22% of the population were born overseas. Of these, 2.5% were born in Greece, making Greek the third most commonly spoken language at home (accounting for approximately 252,200 people, or 1.3% of the population). Greek migration to Australia peaked in the 1950s and 1960s, however the number of new migrants reduced by 17,000 between 1996 and 2006. As a consequence of the steady decline in recent young to middle-aged migrants, the Greek Australian population is ageing at a faster rate than other migrant groups [16]. The 2006 census also revealed that there were 18,380 people born in Cyprus living in Australia, with the largest proportion residing in Victoria (8,400, or 46%). Of these, 65.9% spoke Greek at home. As with Greek migration, there has been a steady decrease in arrivals from Cyprus, and the median age of the Cypriot population in Australia is significantly higher than the total Australian population [17].

In response to the lack of tools to assess foot problems in older people for whom English is a second language, we recently developed and validated a Greek version of the Manchester Foot Pain and Disability Index (MFPDI) [18]. This project involved the assessment of 104 older Greek Australians, who, in addition to completing the MFPDI and the Short-Form 36 (SF-36) health survey, also answered questions related to podiatry needs and underwent a brief clinical assessment of foot problems. The aims of this paper were therefore to: (i) describe the characteristics of foot problems and podiatry needs in this group, and; (ii) to compare the findings to similar studies undertaken in English-speaking older Australians.

## Methods

### Participants

A convenience sample of 104 participants was recruited from four metropolitan Greek-speaking elderly citizen social groups in Melbourne, Australia (three Greek, one Greek Cypriot). Participants were defined as Greek Australians if they were Greek born or descendants. In order to recruit participants, a 10 minute presentation on foot disorders was delivered to each group in Greek. Included in the presentation was a brief outline of the study and a call for volunteers with foot pain to participate. The participants were required to be mobile and capable of walking household distances unaided, in order to evaluate the effect that foot pain has on routine mobility tasks. The study was approved by the Faculty of Health Sciences Human Ethics Committee of La Trobe University (application number: FHEC07/73) and informed consent was obtained from all participants.

### Medical history questionnaire

A questionnaire documenting age, sex, country of birth, living arrangements, medical history, general and foot-specific health-related quality of life and foot problems was administered by an interviewer fluent in Greek (PK). The medical history section of the questionnaire required the participant to state whether they had any of the 15 common conditions listed (including hearing impairment, Parkinson's disease, peripheral vascular disease, leg cramps, diabetes, stroke, cancer, transient ischaemic attack, heart disease/heart attack, high blood pressure, low blood pressure, incontinence, osteoarthritis, rheumatoid arthritis, broken hip). General health-related quality of life was assessed with a validated Greek language version of the SF-36 [19], and foot-specific health-related quality of life was assessed with a Greek language version of the MFPDI [18], provided as additional file 1 (the English version is provided as additional file 2). The total MFPDI score and MFPDI subscale scores were then calculated using the scoring system reported by Garrow et al [20]: none of the time (score = 0), some days (score = 1), on most days/every day (score = 2). Responses to individual items on the MFPDI were also dichotomised by combining the "some days" and "most days/every day" categories. Participants were asked whether they had difficulty finding comfortable shoes, whether they currently received podiatry treatment, and whether they thought they needed more help with their feet. Those who did not receive podiatry treatment were also asked to provide a reason why.

### Clinical foot assessment

The clinical foot assessments were performed by PK, a final year podiatry student. The presence and severity of hallux valgus was determined using the Manchester scale [21]. This instrument consists of standardised photographs of feet with four degrees of hallux valgus - none (score = 0), mild (score = 1), moderate (score = 2) and severe (score = 3) which were matched to the participant's feet. Gradings obtained using this scale are strongly associated with angular deformity measurements obtained from foot x-rays [22,23]. Presence of hyperkeratotic lesions (corns and calluses) were observed and documented. The reliability of these observations when undertaken in older people has been previously established [24].

### Statistical analysis

All statistical tests were conducted using SPSS Release 14 for Windows (SPSS Inc, Chicago, IL, USA). MFPDI scores were considered to be ordinal. Differences in MFPDI total and subscale scores according to sex were determined using Mann-Whitney U tests. Associations between the MFPDI and SF-36 subscale scores were explored using Spearman's *ρ *correlation coefficients. The strength of the correlations was interpreted as follows: none (*ρ *= 0.0 to 0.09), small (*ρ *= 0.1 to 0.3), medium (*ρ *= 0.3 to 0.5) and strong (*ρ *= 0.5 to 1.0). Differences in frequencies of foot problems according to sex, living arrangements (alone or with spouse and/or children) and medical conditions were explored using chi-squared (χ^2^) statistics, as were sex differences in the frequency of the dichotomised MFPDI items.

## Results

### Participant characteristics

Participant characteristics are shown in Table 1, including medical conditions reported by at least 5% of the sample. Most participants were women (n = 64, 61.5%), were born in Greece (n = 59, 56.7%) or Cyprus (n = 41, 39.4%) and lived with their spouse and/or children (n = 88, 84.6%). The most commonly reported medical condition was osteoarthritis (n = 80, 76.9%), and the most commonly reported foot problem was difficulty finding comfortable shoes (n = 39, 37.5%). Only 13 people (12.5%) were currently receiving podiatry treatment, and 42 people (40.4%) stated that they required more help looking after their feet. Of those who did not receive podiatry treatment, the most common reason given were that they did not think their foot problems were severe enough (n = 39, 37.5%), or they managed their foot problems themselves (n = 38, 36.5%). The most commonly observed foot problem was hyperkeratotic lesions (n = 29, 28.8%).

**Table 1:** Participant characteristics


Age (years)	73.0 (5.3)
Sex - n (%)	
Female	64 (61.5)
Male	40 (38.5)
Country of birth - n (%)	
Greece	59 (56.7)
Cyprus	41 (39.4)
Egypt	2 (1.9)
Armenia	1 (1.0)
Australia	1 (1.0)
Education (years)	5.6 (3.4)
Living arrangements - n (%)	
Live alone	16 (15.4)
Live with spouse and/or children	88 (84.6)
Major medical conditions - n (%)	
Heart problems	64 (61.5)
Peripheral vascular disease	60 (57.7)
Osteoarthritis	80 (76.9)
Hips	57 (54.8)
Hands/wrists	41 (39.4)
Spine	39 (37.5)
Knees	24 (23.1)
Feet	9 (8.7)
Cancer	24 (23.1)
Stroke	10 (9.6)
Diabetes	5 (4.8)
Rheumatoid arthritis	5 (4.8)
More than four medications - n (%)	43 (41.3)
Podiatry utilisation/need - n (%)	
Difficulty finding comfortable shoes	39 (37.5)
Currently receives podiatry	13 (12.5)
Reasons given for not attending podiatry (n = 91) - n (%)	39 (37.5)
Foot problems not severe enough	38 (36.5)
Manage feet myself	8 (7.7)
Wasn't aware of podiatry services	2 (1.9)
Too expensive	2 (2.0)
Podiatrist too far away	1 (1.0)
General practitioner manages foot problems	1 (1.0)
Don't like doctors	1 (1.0)
Needs more help looking after feet	42 (40.4)
Foot assessment - n (%)	
Moderate to severe hallux valgus	18 (17.0)
Hyperkeratotic lesions	29 (28.8)

Values are mean (SD) unless otherwise stated

Those who stated that they required more help looking after their feet were more likely to be living alone (χ^2 ^= 6.3, *df *= 1, *p *= 0.024) and have osteoarthritis in their knees (χ^2 ^= 4.9, *df *= 1, *p *= 0.042) or back (χ^2 ^= 4.9, *df *= 1, *p *= 0.028). Those who reported difficulty finding comfortable shoes were more likely to be women (χ^2 ^= 6.2, *df *= 1, *p *= 0.014), and women were more likely to have moderate to severe hallux valgus (χ^2 ^= 9.9, *df *= 1, *p *= 0.019).

### MFPDI subscale scores

Table 2 shows the mean MFPDI subscale scores in men and women. Women were found to have higher MFPDI total and MFPDI subscale scores (*p *< 0.05).

**Table 2:** Median (interquartile range) MFPDI scores according to sex

	Men (n = 40)	Women (n = 64)	*p*
MFPDI - total	9 (5 - 19)	16 (8 - 23)	0.014*
MFPDI - function	6 (4 - 12)	11 (5 - 14)	0.028*
MFPDI - pain	3 (1 - 6)	5 (2 - 8)	0.019*
MFPDI - appearance	0 (0 - 0)	0 (0 - 1)	0.007**

* Mann-Whitney U test significant at *p *< 0.05, ** significant at *p *< 0.01

### Associations between MFPDI and SF-36 subscale scores

Table 3 shows the correlations between the MFPDI and SF-36 scores. There were significant associations for all scores, with the exception of the MFPDI appearance subscale and SF-36 general health subscale. The associations with the SF-36 scores were medium to strong for the MFPDI total score, MFPDI function score and MFPDI pain score, and small to medium for the MFPDI appearance score.

**Table 3:** Correlations (Spearman's ρ) between MFPDI and SF-36 component scores

	SF-36 component scores															
	
	Physical		Role - physical		Bodily pain		General health		Vitality		Social function		Role - emotional		Mental health	
	
	*ρ*	*p*	*ρ*	*p*	*ρ*	*p*	*ρ*	*p*	*ρ*	*p*	*ρ*	*p*	*ρ*	*p*	*ρ*	*p*
MFPDI - total	-0.661	< 0.001	-0.551	< 0.001	-0.681	< 0.001	-0.564	< 0.001	-0.685	< 0.001	-0.539	< 0.001	-0.499	< 0.001	-0.511	< 0.001
MFPDI - function	-0.674	< 0.001	-0.570	<0.001	-0.678	<0.001	-0.570	<0.001	-0.721	<0.001	-0.539	<0.001	-0.520	<0.001	-0.554	<0.001
MFPDI - pain	-0.589	<0.001	-0.439	<0.001	-0.632	<0.001	-0.494	<0.001	-0.561	<0.001	-0.483	<0.001	-0.405	<0.001	-0.389	<0.001
MFPDI - appearance	-0.208	0.035	-0.328	0.001	-0.285	0.003	-0.184	0.062	-0.343	<0.001	-0.255	0.009	-0.256	0.009	-0.195	0.048

### Frequency of dichotomised MFPDI items according to sex

Table 4 shows the frequencies of the dichotomised responses to each of the MFPDI items. The items with the most frequent "some days" or "most days/every day" responses were "I avoid standing for a long time" (78.8%), "I catch the bus or use the car more often" (76.9%) and "I still do everything but with more pain or discomfort" (76%). Women were more likely to respond as "some days" or "most days/every day" for the items "I don't walk in a normal way", "I need help with housework/shopping", "I still do everything but with more pain or discomfort", "I feel self-conscious about my feet" and "I have constant pain in feet" (χ^2^, *p*<0.05).

**Table 4:** Frequencies - n (%) of the dichotomised responses to each of the MFPDI items for the total sample and according to sex

MFPDI item	Total (n = 104)	Men (n = 40)	Women (n = 64)	*p*
I avoid walking outside at all	35 (33.7)	9 (22.5)	26 (40.6)	0.057
I avoid walking distances	72 (69.2)	28 (70.0)	44 (68.8)	0.893
I don't walk in a normal way	52 (50.0)	15 (37.5)	37 (57.8)	0.044*
I walk slowly	68 (65.4)	27 (67.5)	41 (64.1)	0.720
I have to stop and rest my feet	60 (57.7)	19 (47.5)	41 (64.1)	0.096
I avoid hard or rough surfaces where possible	69 (66.3)	22 (55.0)	47 (73.4)	0.053
I avoid standing for a long time	82 (78.8)	29 (72.5)	53 (82.8)	0.210
I catch the bus or use the car more often	80 (76.9)	32 (80.0)	48 (75.0)	0.556
I need help with housework/shopping	39 (37.5)	9 (22.5)	30 (46.9)	0.012*
I still do everything but with more pain or discomfort	79 (76.0)	25 (62.5)	54 (84.4)	0.011*
I get irritable when my feet hurt	64 (61.5)	21 (52.5)	43 (67.2)	0.134
I feel self-conscious about my feet	30 (28.8)	6 (15.0)	24 (37.5)	0.014*
I get self-conscious about the shoes I have to wear	12 (11.5)	2 (5.0)	10 (15.6)	0.099
I have constant pain in feet	68 (65.4)	21 (52.5)	47 (73.4)	0.029*
My feet are worse in the morning	43 (41.3)	17 (42.5)	26 (40.6)	0.850
My feet are more painful in the evening	51 (49.0)	22 (55.0)	29 (45.3)	0.336
I get shooting pains in my feet	63 (60.6)	20 (50.0)	43 (67.2)	0.893

* significant difference between men and women (χ^2^, *p*<0.05)

Note: Responses dichotomised by combining the "some days" and "most days/every day" categories.

## Discussion

The aim of this study was to describe the characteristics of foot problems in a sample of Greek-speaking older people living in Australia. The study findings clearly indicate that foot problems are common in older Greek Australians. In total, the four elderly citizens' clubs had approximately 450 members in attendance on the days participants were recruited. Given that 104 people volunteered, and on the basis of the recruitment criteria, it can be inferred that at least 20% of older people attending these Greek-speaking elderly citizens clubs have disabling foot pain according to the original case definition of the MFPDI. This may be an underestimate, as many older people with foot pain may not have wanted to volunteer for the study. Although our data cannot be considered representative due to the convenience sampling method used, it is interesting to note that the prevalence of disabling foot pain reported here is very similar to population-based studies of community-dwelling older people - generally in the range of 20 to 40% [1,2,4,5].

Consistent with previous studies in other population groups [1,3,6,8,25], older Greek-Australian women appear to have more difficulty with foot problems than men, as they exhibited higher total MFPDI scores, were more likely to report difficulty finding comfortable shoes, and were more likely to have hallux valgus. In a previous study of older people residing in a retirement village, Menz and Morris [26] found that women wore shoes that were shorter, narrower and had a reduced total area compared to their feet than men. Furthermore, wearing shoes substantially narrower than the foot was associated with corns on the toes, hallux valgus deformity and foot pain, wearing shoes wearing shoes shorter than the foot was associated with lesser toe deformity, and wearing shoes with heel elevation greater than 25 mm was associated with hallux valgus and plantar calluses in women [26]. Although we did not assess footwear in this study, it is likely that similar associations would have been evident in this sample, given the higher prevalence of reported difficulty finding comfortable footwear and the higher prevalence of hallux valgus observed in women.

A surprisingly small proportion of the sample were currently receiving podiatry (13%), and of these, the most common reasons given were that they did not consider their foot problems to be severe enough, or they managed their foot problems themselves. Less common reasons provided were limited awareness of podiatry and accessibility or cost barriers. The low rate of consultation in older people with foot problems has been documented in two previous studies conducted in Australia. A survey of 128 older people in New South Wales by Munro et al [27] reported that although 71% of participants reported suffering from foot problems, only 39% had consulted medical personnel about their feet and only 26% identified their foot problems as medical conditions. More recently, the North West Adelaide Health Study found that only 24% of people aged over 65 had accessed podiatry in the previous 12 months [28].

Despite the high prevalence of self-management of foot problems found in this sample, a substantial proportion (40%) nevertheless stated that they needed more help looking after their feet. Our results also highlight that social support and physical ability may influence this need, as this subgroup were more likely to be living alone and to have osteoarthritis in the knees or back. Physical barriers to self-management of foot problems have previously been reported by Campbell et al [29], who found that 63% of older people who had been discharged from podiatry services were unable to care for their own feet due to an inability to bend, hand weakness and poor eyesight, and by Semple et al [30], who found that nearly half of a sample of 30 people with rheumatoid arthritis (mean age 61 years) were unable to self-manage foot problems due to impaired grip strength and difficulty reaching. These findings suggest that although self-management of foot problems has been shown to be effective in older people [31], there are specific subgroups of older people that require professional assistance from foot health specialists.

The findings reported in this survey provide the first insights into the characteristics of foot problems in older Greek Australians using instruments validated in the Greek language. However, these results can only be considered preliminary for several reasons. Firstly, we used convenience sampling of participants from metropolitan Greek elderly citizen social groups. Although such groups are very popular among older Greek-Australians, it is not known whether the characteristics of those who attend differ to those who do not. Secondly, those who participated in the survey may have been motivated to do so for a range of different reasons, and there is little doubt that many older people with foot pain who were in attendance did not volunteer. As such, the sample may not be representative of the broader Greek Australian community. Finally, although inclusion in the survey was limited to those who met the original MFPDI case definition of "disabling" foot pain, it has recently been suggested that this definition identifies virtually all people with foot pain and does not effectively delineate those with disabling symptoms [32]. Therefore, the sample may have included people with very mild or intermittent forms of foot pain.

## Conclusion

This preliminary survey indicates that foot problems appear to be common in older Greek-Australians, have a greater impact on women, and are associated with reduced health-related quality of life. However, only a small proportion of this sample was currently receiving podiatry treatment, and a substantial number stated that they required more help looking after their feet. Further research using the Greek language version of the MFPDI will help identify the foot health needs of this group in more detail, and may assist in improving podiatry service provision for those who have the greatest need.

## Competing interests

HBM is Editor-in-Chief of *Journal of Foot and Ankle Research*. It is journal policy that editors are removed from the peer review and editorial decision making processes for papers they have co-authored. The other authors declare that they have no competing interests.

## Authors' contributions

NF and HBM conceived the study design, PK collected the data, HBM conducted the statistical analysis, and all authors interpreted the results, drafted the manuscript, and read and approved the final manuscript.

## Supplementary Material

Additional file 1 **Greek language version of the MFPDI (first published in Health and Quality of Life Outcomes, 2008;6:39)**. Click here for file

Additional file 2 **English language version of the MFPDI**. Click here for file
