# Correction: A survey of foot problems in community-dwelling older Greek Australians

**DOI:** 10.1186/1757-1146-6-13

**Published:** 2013-04-05

**Authors:** Patricia Kaoulla, Nicoletta Frescos, Hylton B Menz

**Affiliations:** 1 Department of Podiatry, Faculty of Health Sciences, La Trobe University, Bundoora, Victoria, Australia; 2 Musculoskeletal Research Centre, Faculty of Health Sciences, La Trobe University, Bundoora, Victoria, Australia

## Correction

Since the publication of our Research article [1], the copyright of the Manchester Foot Pain and Disability Index (MFPDI) now states a licence agreement is required for publication. As a result, Additional file 2 has been removed, the English language version of the MFPDI can be found through the ISIS Outcomes website [2].
