# Supplementary material for: A survey of foot problems in community-dwelling older Greek Australians
Source: J Foot Ankle Res. 2011 Oct 20;4:23. doi: 10.1186/1757-1146-4-23 (PMC3212908; doi:10.1186/1757-1146-4-23)
Supplement: Additional file 1 — Greek language version of the MFPDI (first published in Health and Quality of Life Outcomes, 2008;6:39). [file 1757-1146-4-23-S1.PDF]

MANCHESTER FOOT PAIN AND DISABILITY INDEX – GREEK VERSION

Παρακάτω υπάρχουν μερικές παρατηρήσεις για προβλήματα που παρουσιάζονται στους ανθρώπους λόγω **πόνου στα πόδια τους.**

Για κάθε παρατήρηση σημειώστε αν αυτο σας έχει συμβεί τον **περασμένο μήνα**. Αν ναι, συνέβει μόνο μερικές μέρες, τις περισσότερες ή κάθε μέρα του περασμένου μηνα;

Σας παρακαλώ να σημειώσται σε ένα κουτί για κάθε πρόταση

	**Ποτέ**	**Μερικές μέρες**	**Τις περισσότερες / Κάθε μέρα**	
**Εξαιτίας του πόνου στα πόδια μου**				
Αποφεύγω να περπατώ έξω εντελώς	❑	❑	❑	
Αποφεύγω να περπατώ μακρινές αποστάσεις	❑	❑	❑	
Δεν περπατώ με κανονικό τρόπο	❑	❑	❑	
Περπατώ σιγανά	❑	❑	❑	
Πρέπει να σταματήσω και να ξεκουράσω τα πόδια μου	❑	❑	❑	
Αποφεύγω να περπατώ σε σκληρές ή ανώμαλες επιφάνειες	❑	❑	❑	
**Εξαιτίας του πόνου στα πόδια μου**				
Αποφεύγω να στέκομαι για πολυ ώρα	❑	❑	❑	
Παίρνω το λεωφορείο ή χρησιμοποιώ το αυτοκίνητο πιο συχνά	❑	❑	❑	
Χρειάζομαι βοήθεια με τις δουλειές του σπιτιού/με τα ψώνια	❑	❑	❑	
Ακόμα κάνω τα πάντα αλλά με περισσότερο πόνο ή ενοχλήσεις	❑	❑	❑	
Γίνομαι νευρικός όταν πονάνε τα πόδια μου	❑	❑	❑	
Ντρέπομαι για τα πόδια μου	❑	❑	❑	
Ντρέπομαι για τα παπούτσια που πρέπει να φορώ.	❑	❑	❑	
Έχω συνέχεια πόνο στα πόδια μου	❑	❑	❑	
Τα πόδια μου είναι χειρότερα το πρωί	❑	❑	❑	
Τα πόδια μου πονούν περισσότερο το βράδυ	❑	❑	❑	
Με σουβλίζουν τα πόδια μου	❑	❑	❑	
**Εξαιτίας του πόνου στα πόδια μου**				Δεν ισχεί
Αδύνατω να τα βγάλω πέρα την προηγούμενη δουλειά μου	❑	❑	❑	❑
Δεν κάνω πια τις προηγούμενες δραστηριότητές μου (αθλήματα, χορό, περπάτημα σε λόφους κτλ)	❑	❑	❑	❑

Σημειώστε εδώ όταν έχετε διαβάσει όλες τις προτάσεις σε αυτή τη σελίδα ❑
