# Supplementary material for: A survey of foot problems in community-dwelling older Greek Australians
Source: J Foot Ankle Res. 2011 Oct 20;4:23. doi: 10.1186/1757-1146-4-23 (PMC3212908; doi:10.1186/1757-1146-4-23)
Supplement: Additional file 2 — English language version of the MFPDI. [file 1757-1146-4-23-S2.PDF]

MANCHESTER FOOT PAIN AND DISABILITY INDEX – ENGLISH VERSION

Below are some statements about problems people have because of **pain in their feet.**

For each statement indicate if this has applied to you during the **past month**.

If so, was this only on some days or on most or every day in the past month?

	None of the time	On some days	On most/every day/s	
**Because of pain in my feet:**				
I avoid walking outside at all	❑	❑	❑	
I avoid walking long distances	❑	❑	❑	
I don’t walk in a normal way	❑	❑	❑	
I walk slowly	❑	❑	❑	
I have to stop and rest my feet	❑	❑	❑	
I avoid hard or rough surfaces when possible	❑	❑	❑	
**Because of pain in my feet:**				
I avoid standing for a long time	❑	❑	❑	
I catch the bus or use the car more often	❑	❑	❑	
I need help with housework / shopping	❑	❑	❑	
I still do everything but with more pain or discomfort	❑	❑	❑	
I get irritable when my feet hurt	❑	❑	❑	
I feel self-conscious about my feet	❑	❑	❑	
I get self-conscious about the shoes I have to wear	❑	❑	❑	
I have constant pain in my feet	❑	❑	❑	
My feet are worse in the morning	❑	❑	❑	
My feet are more painful in the evening	❑	❑	❑	
I get shooting pains in my feet	❑	❑	❑	
**Because of pain in my feet:**				**Not applicable**
I am unable to carry out my previous work	❑	❑	❑	❑
I no longer do all my previous activities (sport, dancing, hill-walking, etc)	❑	❑	❑	❑

Tick here when you have read all the statements on this page ❑
